# Shared Construction of Social Pretend Play Sequences at the Kindergarten

**DOI:** 10.5964/ejop.12443

**Published:** 2024-05-29

**Authors:** Valentina Fantasia, Francesca Moncalli, Arianna Bello

**Affiliations:** 1 Department of Philosophy and Cognitive Science, Lund University, Lund, Sweden; 2 Department of Education, Roma Tre University, Rome, Italy; Tampere University, Tampere, Finnland

**Keywords:** social pretend play, kindergarten children, sequential analyses, peer interactions

## Abstract

Pretend play is usually defined as an activity wherein objects and actions (but also affective expression, at times) are separated from their original meanings. Its developmental appearance is set around the second year of life, and increases dramatically in duration, frequency and quality when play episodes start becoming more complex, both linguistically and interactionally reaching its peak in preschool years. To date, however, little attention has been paid to how social pretend play emerges and develops before the age of three. Our study aims to investigate early spontaneous pretend play interactions between children aged 19 to 28 months attending the same kindergarten. We used micro-analytical coding of video-recorded interactions to explore sequences of interaction where children coordinated their actions to engage in social pretend play with objects. Our analyses showed that co-constructed sequences appeared organised by a turn-alternation structure already at 19 months, and children used embodied and material resources afforded by the sequential organisation of actions to dynamically manage their participation. Although explorative, our results seem in line with previous reports suggesting an early onset of social pretend play developing over a continuum from being predominately an individual activity to progressively becoming a co-constructed endeavour.

Children’s play has been a flourishing area of interest for developmental psychologists and early childhood educators (Garvey, 1990), and despite recent critical discussions (see Lillard et al., 2013 for a review), scientists largely agree that play, in almost all its different forms, supports and creates unique developmental opportunities in socio-cognitive, motor, affective and communicative arenas (Christie, 2022; Sutton-Smith, 1981; Taylor, 2013). Recently, social pretend play has occupied intense research interest. Pretend play is usually defined as an activity wherein objects and actions (Piaget, 1945) are separated from their original meanings. Its developmental appearance is set around the second year of life, and increases dramatically in duration, frequency and quality when play episodes start becoming more complex, both linguistically and interactionally reaching its peak in preschool years (Smith & Lillard, 2012). Studies within this domain have shed light on the early origin and development of children’s participation in social interactions where pretence is a joint endeavour, searching for constitutive aspects such as, among others, children’s interactional and communicative competence type of relationship among co-participants, attentional capacities and familiarity with play practices. However, research investigating the early emergence of social pretend play among pre-school children is not extensive (Fantasia & Nomikou, 2022) and mostly committed towards identifying the progression of social pretend play forms, and their correlation with children’s developing abilities (Lillard et al., 2011; Thompson & Goldstein, 2022). The present work is a preliminary exploration of such issues. We endorsed an ethnomethodological approach to look at young children’s (19- to 28-months old) spontaneous engagement in social pretend play interactions within different pretence settings at kindergarten. By adapting analytical categories previously used by Monaco and Pontecorvo (2010), we analysed how interactional sequences of social pretend play are coordinated and co-constructed.

## Pretend Play Interactions as Meaning-Making Practices

Social pretend play, also referred to as imaginative, symbolic, or fantasy play, is commonly defined as an activity involving some degree of taking an “as-if” stance between two or more playing partners (Bateson, 1955). Pretend play is an exquisitely ‘event-generative’ (Sacks, 1995, p. 496) phenomenon, wherein children actively and creatively appropriate aspects from the adult world to create new rules for new activities and scenarios (Breathnach et al., 2018). Social pretend play in school children often takes the form of sharing imaginary scenarios, e.g., through storytelling, or crafting narratives with invented characters and settings (Theobald et al., 2022). By doing so, children create new cultural practices embedded into an inter-subjective context (Gaskins, 2013), where relational dynamics, conflict resolution and generation of conflicts occur and need to be managed (Breathnach et al., 2018). Mastering verbal competence plays a key role in the extent to which imaginary scenarios can be rich or complex; solitary or shared with others. The use of symbolic features in storytelling or during a shared construction of playing scripts may be considered an evolved form of communication or meta-communication (Giffin, 1984). Language competence also influences how young children (before 3 years of age) successfully initiate or sustain social play interactions with peers (Luo et al., 2022).

Nevertheless, some key features of pretence may be visible long before children master communicative skills (Göncü & Gaskins, 2011). Long before they enjoy engaging in complex, narratively articulated imaginary play interactions children engage in imaginary interactions in their family environment, e.g., with siblings (Fantasia & Nomikou, 2022; Howe & Leach, 2018), beginning to use objects symbolically or functionally in many ways, which vary in degree of abstraction/iconicity (Rubin et al., 1978). From infancy, early social play is grounded in the caregivers’ capacities to re-use infants’ embodied displays to organise shared patterns of play (Fantasia et al., 2014). At 10 months infants can predict and anticipate the interactive structure of teasing and pretence-like activities, such as peak-a-boo (Bruner & Sherwood, 1976) and around 13 months they engage in simple forms of complementary play interactions with peers (Howes & Matheson, 1992). At 15 months of age, toddlers seem already capable of initiating simple pretend instances by socially coordinating embodied resources, e.g., using toys or objects that are immediately available in the physical space, through the supported actions of the caregiver (Filipi, 2022). Early instances of pretending may involve reproducing everyday actions at home using objects that afford specific functions or actions (e.g., a salad bowl is used as a cooking pot); or creatively deploying functional objects in arbitrary ways (e.g., when a twig becomes a sword). Progressively over development, social pretence becomes a multi-party endeavour, moving from individual functional use of toys to socially richer forms of play with different degrees of coordination and reciprocity among participants (Howes & Matheson, 1992), where the quality of pretend play seems to evolve as a function of their relationships (Luo et al., 2022). The pretend “gradient” increases over developmental time: during preschool years (that is, before 3 years), pretend forms progressively change becoming more abstract, to include “invisible objects” living entirely in the child’s imagination (Göncü & Gaskins, 2007). Objects—real ones or imaginary—shape the quality of what is being played as well as the participatory possibilities among playing partners in shared activities (Cobb-Moore et al., 2009; Houen & Danby, 2021). To our knowledge, however, no research has investigated the sequential organisation of early social play, that is, how young children initiate and maintain a reciprocal and shared orientation towards a pretend play activity; and how children’s spatial positioning enables, crafts, affords specific forms of participation in various activities and settings.

## Social Interactions at the Kindergarten

Forms and possibilities for participating in co-constructed sequences of shared actions (and goals) are manifold in the ecology of infants’ daily social interactions (Lerner et al., 2011). Infants are gradually drawn into conventionalised forms of interaction through repetitive daily experiences of play. In social routines, they learn to recognise complex interactional formats, and perform particular actions contingently within an action sequence (Fantasia et al., 2014; Ratner & Bruner, 1978). Early playful interactions with meaningful adults are contexts in which infants learn how to participate, before developing an understanding of the real and the imagined, just by learning what to do when and how to respond (Nomikou et al., 2017). In the ecology of their daily social encounters, e.g., at kindergarten, or in interaction with adults and siblings, young children make use of environmental resources (e.g., space, objects) to accomplish purposes while adapting to relevant contingencies (Kern, 2023; Ochs, 1990). Early educational settings, such as kindergartens, represent social arenas to experiment rich with opportunities for multifaceted arrangements with others (Evaldsson & Corsaro, 1998; Theobald, 2009). As such, they are privileged contexts to observe the ecological development of children’s social practices and experiences, including play, as they afford the observation of social exchanges and interactions with peers and adults. Children’s participation in social activities within those settings is organised and dynamically orchestrated by different interactional strategies (Fatigante et al., 2022; Monaco & Pontecorvo, 2010), locally designed to accomplish and manage a variety of purposes and situations. For instance, young children use aspects of sequential organisation design (e.g., pre-sequences) aimed at recruiting and securing attention and availability of potential co-participants in shared play (Burdelski & Cekaite, 2022).

Our study seeks to provide preliminary evidence of the early construction of shared pretend play practices in a group of young children (from 19- to 28- months old) attending the same kindergarten. We endorsed an ethnomethodological approach to analyse the temporal unfolding of interactional sequences of interaction where children coordinated their actions to engage in social pretend play with objects, moment-to-moment.

## Method

### Participants

Fourteen children with typical development, between 19 to 28 months (*M* = 9.3; *SD* = 4.4; *N* = 10, 4 females, Table 1) participated in the study. Children attended the same kindergarten near Parma, Italy. In line with the aims of the study, and to facilitate the emergence (and observation) of spontaneous play sequences with as little interference as possible, children were divided into ad-hoc subgroups, as follows:

- “Small” group, composed of one boy and one girl, mean age 19 months
- “Young” group A, composed of 2 boys and 2 girls, mean age 24.7 months
- “Young” group B, composed of 3 boys and 1 girl, mean age 24.7 months

**Table 1:** Participants Demographic Information by Group

Group	Child name	Age (months)	Sex
Small	Alfie	19	M
Small	Emily	19	F
Young A	Marco	28	M
Young A	Edo	22	M
Young A	Lea	23	F
Young A	Giovanna	26	F
Young B	Tommy	24	M
Young B	Lorenzo	24	M
Young B	Matilde	28	F
Young B	Francesco	23	M

Children were allocated to subgroups by teachers according to the following criteria 1) their affinity/relationship with each other (preferred); and 2) their ages and sex, in order to have balanced male-female ration and matched ages within the same group.

### Setting

**Figure 1:**
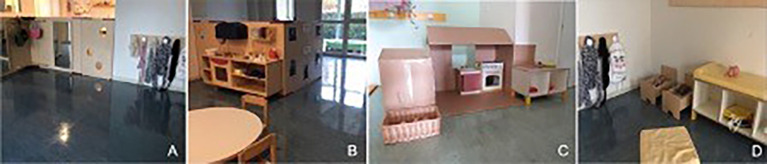
Pretend Play Settings *Note*. A = Clothing/Dressing up; B = Young group kitchenette; C = Small group kitchenette; D = Nursery/Care.

The study received ethical approval from the ethics committee of Roma Tre University. Data were collected at a small kindergarten near Parma, norther Italy. Observations of play episodes were conducted in three different play settings of the kindergarten, presenting pretend accessories and facilities (see Figure 1): Two kitchenette areas, one nursery/care area and one area with clothing/dressing up accessories.

Children were observed during sessions of spontaneous play in small groups inside the kindergarten. All observation sessions were video recorded by one teacher, with a camera placed on a tripod at a distance of around 2 metres from the space in which the children were playing, covering a 180-degree angle. The teacher was slightly removed from the play space, “focused’ on observing what was happening and filling out an ad hoc observation grid.

Prior to the beginning of the study, caregivers of all participating children were informed of the study purposes, and consent forms (to take part in the study and to use data for scientific dissemination purposes) were collected. Only children whose caregivers gave written consent to participate were included in the study. Participants sensitive data, including signed consent forms, were anonymised, encrypted and safely stored. For analytic purposes, each child has been assigned with an ID and a fictional name.

### Procedure

Each group was observed individually once a week, for a total of 4 weeks observation for each group. Prior to the beginning of each observation session, children of the same group were picked up from the classroom by one teacher and accompanied to the room/setting selected for the video-observation during that week. Groups alternated within weeks and rooms, so that each group was observed in each room/setting at least once. As they were brought into the selected play setting, children were allowed to play freely without any adult intervention, for about 15–20 minutes. Then, they were taken back to their classroom.

### Data Analyses

Our data corpus consisted of 12 filmed data sessions, four for each group, for a total of 02:25:49 hrs videos. Following a preliminary observation phase, we chose to focus our analyses on how children use local resources (both behavioural as well as materials) to *co-construct sequences of pretend play.*

Co-constructed sequences were identified as displays of individual behaviours coordinated into turn-shaped sequences, where children 1) initiated a new, shared play activity where each child employs an object and/or proposes a new course of action; or 2) complemented or joins in another child’s ongoing action, thereby creating a new interactional event. Qualitative analyses were carried out by using ELAN (Version 6.5; The Language Archive, 2023). Individual behaviours, as movements, gazes, postures and actions as they are continuously produced in spontaneous play interactions were treated as visible displays of orientation and participation (Goffman, 1988), and coded for each child in each data session. For the purposes of the study, behavioural data on frequency and durations in the “Young” groups (A+B; age-matched) were combined into one single age group named “Young”. After micro-analysing children’s individual behaviour, we shifted our focus on macro-descriptions of sequences of pretend play, involving the co-presence of two or more children, in regard to specific interactional aspects (defined below as analytical and interpretative categories). Macro-descriptions focused on how children co-constructed locally-crafted and situated sequences of play, by incorporating other’s moves and actions. Four analytical and interpretative categories were used to identify and describe children’s behaviours in social play sequences. Categories were adapted after the work by Monaco and Pontecorvo (2010), resulting as it follows:

1. *Co-construction of the play activity*: how behaviours and material resources are coordinated and shared to construct multiparty play sequences.
2. *Participants’ spatial positioning*: toddlers’ spatial and bodily orientation while interacting.
3. *Participatory role(s):* conceiving participation as a dynamical process, we looked at the displays of interactional engagement and changes in children’s participation over time. According to the specific interactional situation, children can flexibly move from one participatory role to another, e.g., from active forms of participation to more peripheral ones within the same play sequence.
4. *Sequential organisation of co-constructed play episodes:* how participants manage turn-taking and alternation. A turn is intended here as a single action displayed towards a clear end-goal.

In the next section we present results on macro-descriptions of play sequences according to the categories/interactional aspects outlined above. For each analytical and interpretative category (see above), we also included micro-analytical descriptions of selected play sequences depicting relevant aspects of each category. Although our study has no developmental comparative purposes, we report our findings separately for the two age groups observed.

## Results

### The Shape of Pretend Play Episodes at the Kindergarten

In order to investigate the co-construction of social play sequences, selected episodes of joint play sequences were analysed in their sequential unfolding, from the timeframe marking the end of each child’s individual activity to the moment in which the joint activity was concluded. Overall, sequences built over multiple interactional turns allowed for increasingly longer and richer play exchanges, as each new turn created a new interactional affordance and the possibility for a new object to be incorporated in the ongoing play scenario. For example, a sequence that began around a table, with one child putting food on a plate, was shortly followed by the participation of two more children who added further play items.

In line with children’s age-matched competences, children’s play was mostly functional in all. That is, available play items and facilities were employed for their pretend function, and no imaginary or creative scenario was verbally created around the toy(s) in use. Dolls were used to practice nappy changing, but not for displaying hugging, caressing, or any affective engagement. This was not surprising, especially since children’s communicative capacities were largely limited, and mostly based on non-verbal resources, such as reciprocal bodily orientation while using the same item(s), and gaze alternation between actions, items and co-participant(s). In both age groups children displayed a very limited range of gestures (mostly deictic). Along the same line, an additional interesting aspect emerged: children’s play efforts were mostly oriented towards exploiting the functional quality of objects used rather than enacting their symbolic play potential. That is: They engaged attentively and for the longest time on *preparing a pretend meal, but they would not go on eating what they had just prepared*. Preparing meals, choosing among different toy foods to put inside and setting the table constituted the core of the play activity. Once these action sequences were concluded, the children did not pretend to eat lunch together, as one might have expected, but moved on to do something else.

### Co-Construction of the Play Activity: How Behaviours and Material Resources are Coordinated and Shared

Children spent most of their playing time by themselves, occupied with objects available in the play area. They often stood close to each other, playing on their own but sharing their peripheral space and play facilities (e.g., a toy kitchen; a wide round table; a deep and high closet with dressing-up clothes to hide within). Very often, sharing spaces accidentally resulted in crossing each other’s ongoing activity, creating opportunities for children to engage in episodes of co-constructed play sequences, wherein they complemented/supported the other’s action in progress. In the Small group the co-constructed play sequences observed are less frequent and shorter than those displayed by older children.

Although individual play was the predominant modality in both groups, older children engaged in negotiations about how and when to use resources (objects, spaces, and different furniture present in each room) through the combination of multimodal resources such as gaze, posture and position. Participatory opportunities were afforded by the co-presence of other children standing and playing nearby: by observing and/or imitating others’ play practices; by using objects in new, unexplored ways; by initiating new dyadic play sequences and experiencing new ways of participating in participation frameworks, e.g., by negotiating objects or spaces to play. Object use varied and changed as children engaged in different participatory configurations and action initiatives, shaping the interactional unfolding.

**Figure 2:**
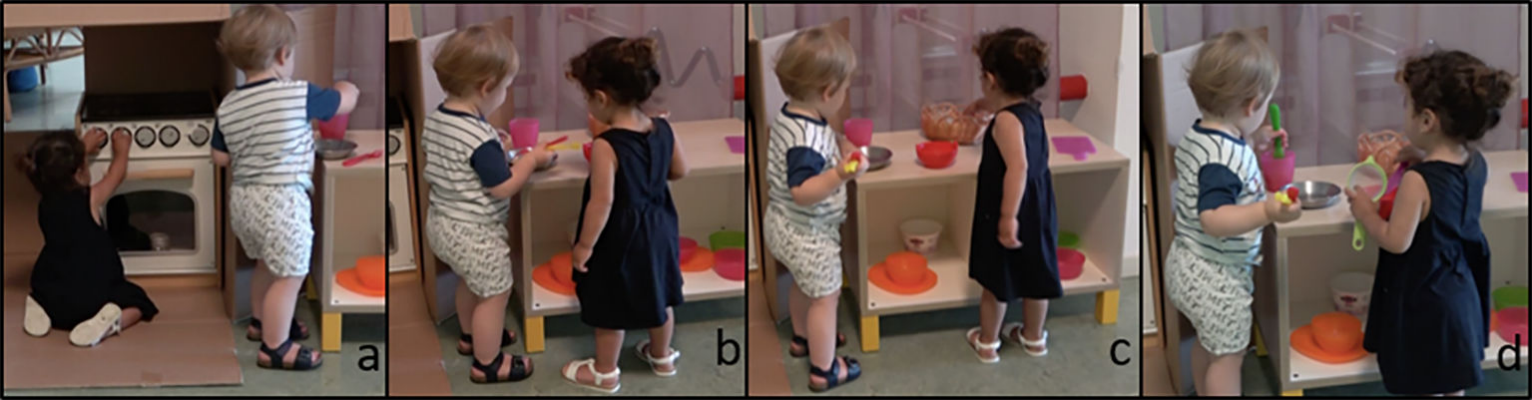
Emily and Alfie Playing at the Kitchenette

In the example below, Emily (E) and Alfie (A; both 19-months old) are engaged in a long, uninterrupted episode of play in the kitchenette (approximate duration 4:45 minutes). Each of them is focused on pretend cooking in a different part of the play setting (Figure 2a). About halfway through the ongoing play, E gets closer to A to observe his playing activity (Figure 2b), but she does not intervene or take any initiative. Before returning to play, E fetches a new object from the basket (Figure 2c) and then shifts her gaze from A’s action to his face (Figure 2d), as to monitor his reaction. A does not reciprocate the gaze, continuing with his own activity. Then E sets her pretend cooking next to A, playing in parallel. During the entire play episode there is no display of reciprocal gaze among the two children, as they do not look at each other simultaneously.

### Participants’ Spatial Positioning

Toddlers’ use of space and bodily orientation while interacting varied across ages. While individual play interactions with objects remained a preferred modality, play spaces became increasingly more shared as children tended to move fluidly across the play settings, often following the other’s movements and positions. In the group of older children, although the most common play mode was solitary play with objects, spatial configurations seemed organised towards seeking co-presence: As a child moved from one play area to another within the room, another child (or more than one) gradually followed him/her. As a result, spatial positioning around the kitchenette, the dining table, or the nursery changing facility was not static but dynamically re-arranged as children moved across the room to engage in new individual and shared play activities. Fluid spatial positioning afforded an array of participatory configurations (Kendon, 1973) ranging from children engaged in prolonged solitary play in parallel to others’ activities, to standing on the side observing others’ play.

Sharing spaces enabled children’s observation of, or “unplanned” engagement with others’ play activities, which progressively led the way towards play activities involving new participatory frameworks. Pretence cooking actions at the kitchenette, for instance, became progressively more coordinated: Children did not simply cross over each other’s action around the stove or with the toy food; they progressively placed and merged bits of individual actions into others’ individual action sequences. Towards the end of the third session, for example, Lea (L) and Giovanna (G) engaged in a brief sequence of coordinated actions: As L engaged in a long sequence of putting toy vegetables into a saucepan on the stove, G moved closer to L. She remained still, observing L’s movements, for almost 4s. Then, very quickly G fetched a tomato from the shelf and placed it into L’s saucepan. As L continued to add more vegetable and stir the saucepan, G then got hold of a stirring stick and started stirring vegetables in the pan, clashing her stick with that of L. The joint stirring sequence went on for almost one minute.

**Figure 3:**
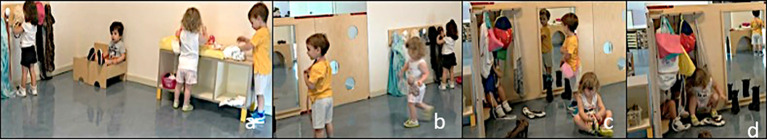
Children’s Spatial Positioning Varied According to the Others’ Position in the Room

Below (Figure 3) another example of how children moved across the room(s) changing their spatial positioning (and therefore, activities) with some reference to the others’ position is presented. As they enter the room, all children (Young group) start playing in the nursery/care area of the room (Figure 3a). After approximately 10 minutes, Edo (L, yellow t-shirt) moves towards the opposite side of the room, in the dressing-up area, followed by Lea (L, with white trousers; Figure 3b). A few minutes later, Giovanna (G, black trousers) moves in the area as well to go and hide in the closet (Figure 3c), followed by L, who was initially playing trying shoes opposite to G (they were not oriented towards each other). L interrupts her ongoing shoe-trying activity to go and sit next to G in the closet (Figure 3d).

### Participatory Role(s)

Positions and roles were activity-related. When children stood close to each other to play with the same toy, or when they contributed to the same activity by placing bids of actions in sequence, they flexibly moved from one participatory role to another, e.g., from active forms of participation to more peripheral ones, even within the same play sequence. Most of the children engaged in co-constructed sequences by taking different roles, such as “initiators”, or “bystanders”. In the Young groups, two children often acted as joint actions-propulsors, as they initiated new action sequences by offering toys to another participant, or by joining in ongoing play interactions.

**Figure 4:**
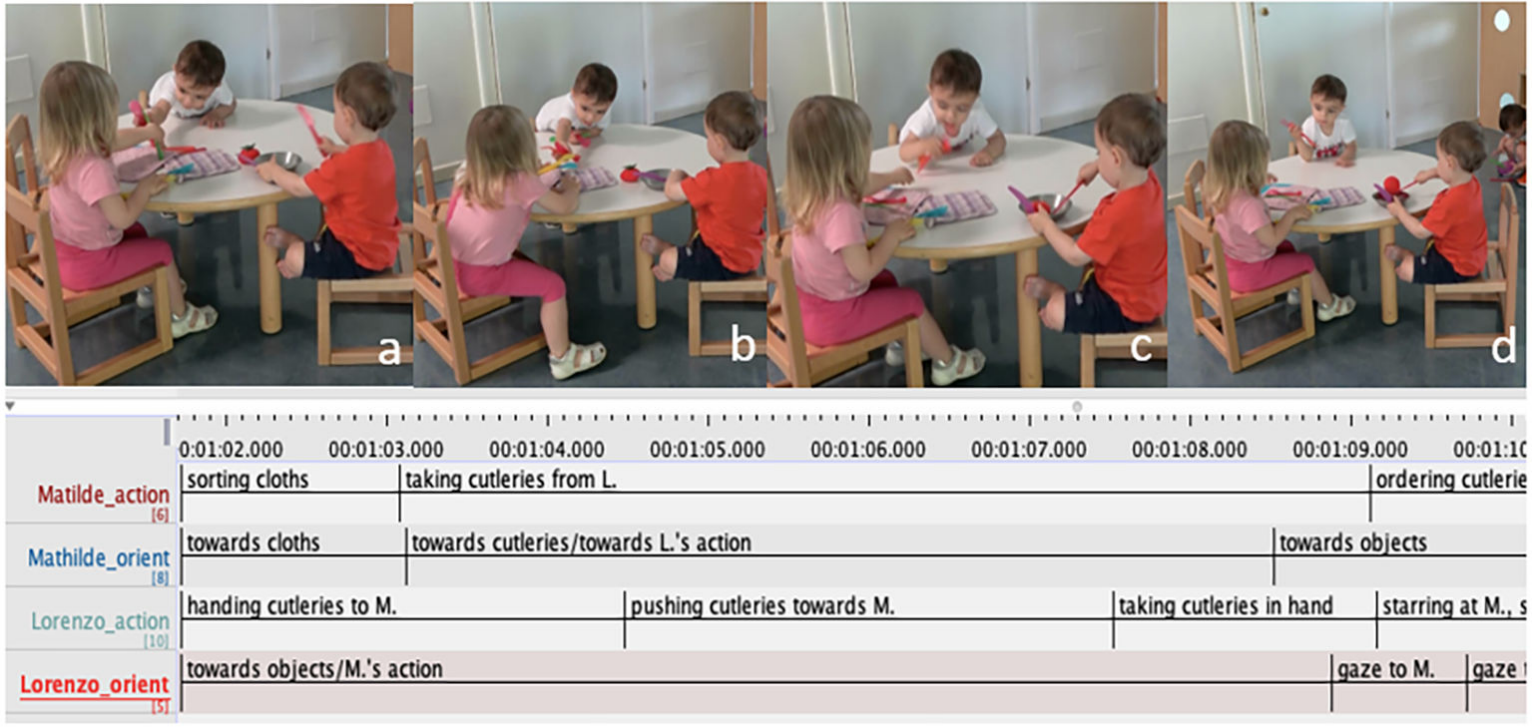
Lorenzo Intermittently Moves in and off the Ongoing Activity by Matilde, Including Positioning Plastic Cutleries on Cloths as to Pretending Setting the Table

In the episode below, one child offers toy cutlery to another child twice, resulting in an extended sequence of co-constructed pretend cooking (Figure 4). In this episode, Lorenzo (L, white t-shirt), Matilde (M, pink t-shirt) and Francesco (F, red t-shirt) are playing at the table in the kitchenette area, each playing on their own. M is playing with cloths and arranging toy cutlery, L holds a few items of toy cutlery and Francesco is pretending to cook and eat a tomato in a small plate. At the beginning of the episode L, who was holding some toy cutlery (two forks and a knife), moves forward towards M, outstretching his arms to offer her the toy knife (Figure 4a). M, who is sitting next to him playing with kitchen cloths (Figure 4a), turns towards Lorenzo and grabs the objects. Then as L puts two toy forks on the table, he starts pushing them towards M (Figure 4b). When she tries to grab the forks, L quickly takes the objects back while monitoring the ongoing activity at the table (Figure 4c). A few seconds later, he orients his body towards M and glances shortly at her face– the first time since the beginning of the episode, as to check on her reaction (Figure 4d). M does not look at L but continues setting cutlery on the cloth. About ten seconds later all three children leave the table.

As they entered the selected playing areas, some children (particularly in the Young groups) raced to get as many objects as they could, while others remained still, waiting until the ground was clearer or taking an observational stance before starting playing. This shaped the type and frequency of opportunities for participating in social activities: having more objects enabled children to construct more varied play activities and invited more opportunities to engage in shared play. Older children displayed an early ability to manage participatory frameworks by selecting objects and moving within spaces in a way that facilitated or restricted others’ activity. Objects were recurrently moved in positions that only some children had access to but not others: dolls, a particularly popular play object among children, were often hidden behind the baby cot or the table so that no one could play with them except the ‘hider’. Children in the Young group often recruited co-players to initiate a new play sequence: They offered objects, e.g., by pushing toys towards another child while seated around the table, then waited a few seconds to see how the other responded. Or by getting closer and making preliminary attempts to join in the ongoing activity of another child, by, e.g., gently touching the toy in her hands.

### Sequential Organisation of Co-Constructed Play Episodes

We focused on how children managed and supported turn-taking when a new action sequence was established. Children’s interactional sequences appeared as organised into several small-steps of actions resulting in longer macro-sequences, e.g., playing with putting the doll to sleep in the cot, putting on its pyjamas, laying it down in the cot and pulling the blankets over it. In both age-matched groups, children predominantly employed non-verbal resources for initiating, coordinating or closing an interactional sequence, with greater use of embodied markers of co-orientation towards the ongoing activity, such as seeking spatially orienting of the body in a face-to-face position, or leaning forward on the table as they sought to share objects with a distant partner, sitting at the opposite side of the table. Children made a limited use of gaze behaviours to manage the interaction, e.g., as an attention-getting device. Gaze patterns, such as alternating gaze from object to person, or modulation of length and frequency of gazes in social exchanges, were mostly deployed by 22–28 months old children when engaged in interactional sequences involving the joint coordination of objects. Younger children directed their gaze behaviour mostly towards their co-interactants’ ongoing activity (e.g., their hands or any object therein).

When another child joined in with a new action, pauses between each turn became longer, resulting in longer sequences of co-constructed play. For example, once the doll was placed into the cot by one child, another child (who was standing close by, observing) holding a cloth waited for the doll to be positioned and then placed the cloth over the doll. This type of sequential co-construction also afforded longer pauses between one turn and the other. Pauses and action turns were rarely managed by children through vocal resources (it only occurred once, w), but mostly through gaze patterns to reciprocally monitor the other’s engagement in the ongoing activity and situated actions. When one participant responded with a specific kind of action, “some analysis, understanding or appreciation of the prior turn will be displayed in the recipient’s next turn of talk” (Heritage & Atkinson, 1984, p. 255). Co-interactants often waited for the other's action to be almost completed before placing a new action, resulting in longer play episodes and engagements. On the contrary, in the youngest group children engage in shorter episodes constructed over quick turn alternation and brief pauses.

**Figure 5:**
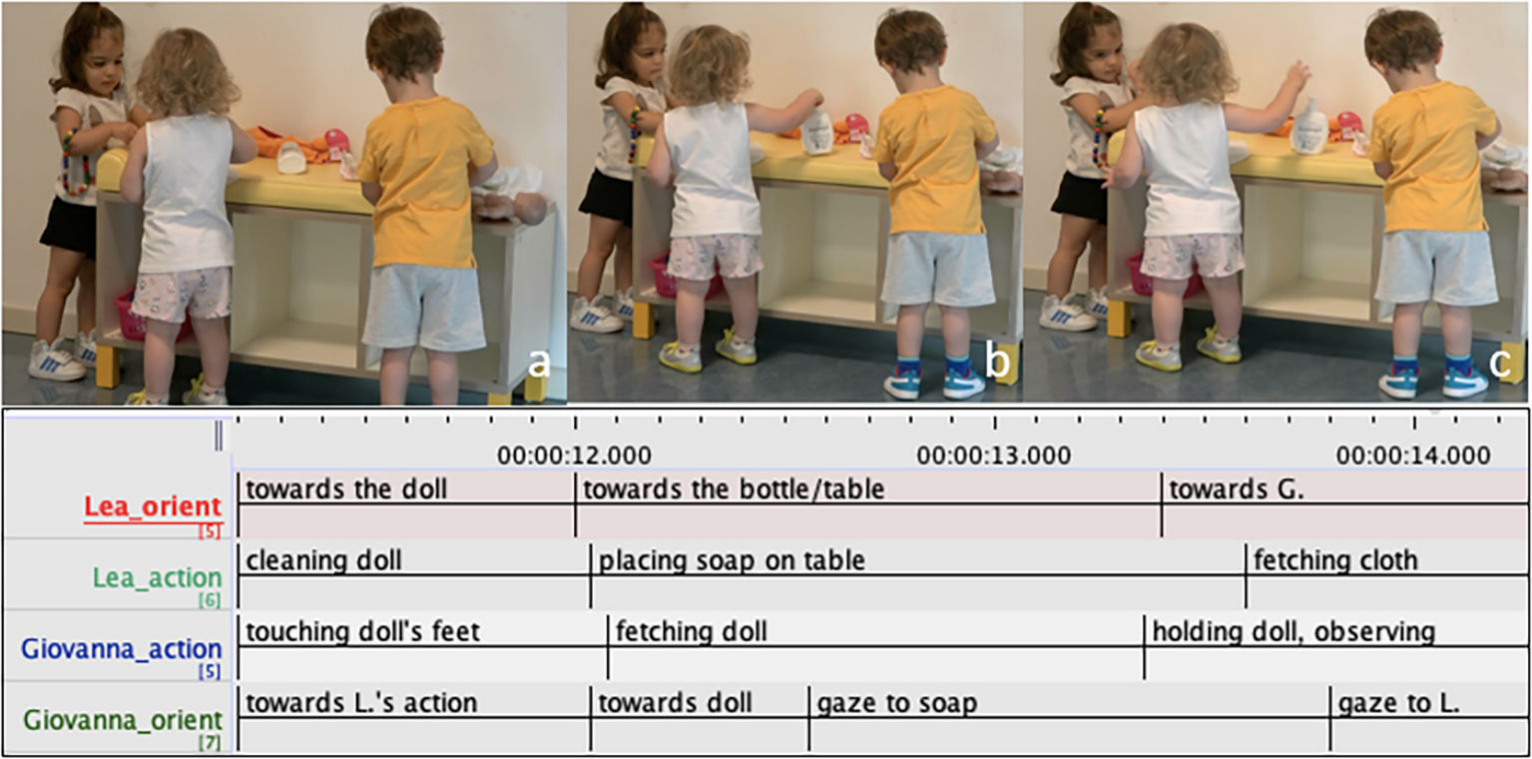
Co-Construction of the Play Sequence, Part 1: Giovanna Takes the Doll Away From Lea as she Shifts her Attention From the Doll to the Soap Bottle

The episodes described below present two extracts from a long nappy-changing sequence shared among three children. In the first extract (Figure 5) Giovanna, Lea and Edo are playing at the changing facility in the nursery area. L (face not visible) is engaged in cleaning and changing a doll with a doll at the left side of the table, E is doing the same at the opposite side and G is oriented towards L’s activity. While L is playing with the doll, G begins touching the doll’s feet (Figure 5a). A few second later L turns slightly to the right to place the soap bottle on the table (Figure 5b), orienting her attention away from the doll. As this happens, G first looks at the doll and then quickly fetches L’s doll (00:12.08). L does not immediately acknowledge this move by G, maintaining her gaze and posture oriented towards positioning the soap on the table (Figure 5b). As G holds the doll, her gaze moves first towards L’s ongoing action (00:12.56), and then briefly towards L’s face (Figure 5c), as to monitor her reaction. L then looks towards G, and slowly moves away from the table holding the cloth.

**Figure 6:**
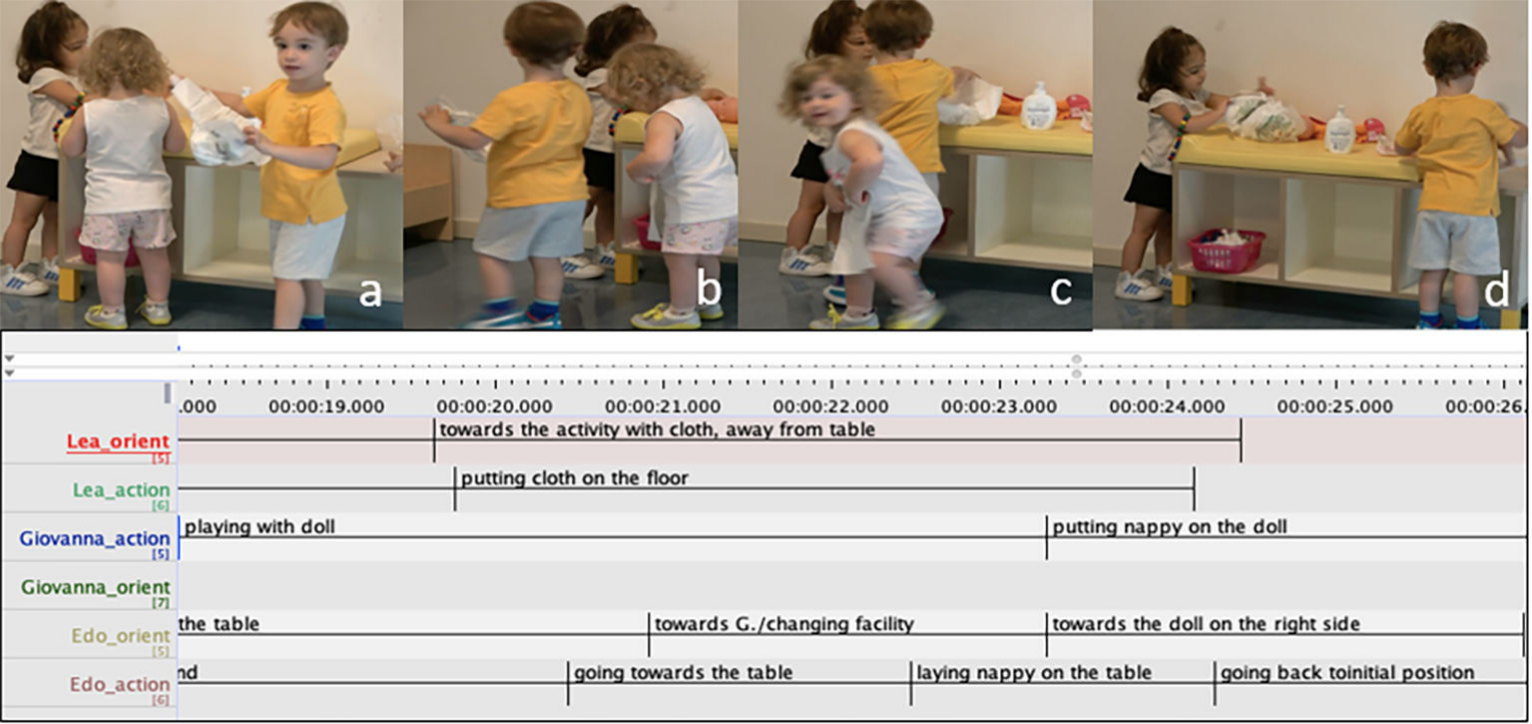
Co-Construction of the Play Sequence, Part 2: When the Space in the Changing Table Clears, Edo Lays Down a Diaper Next to Giovanna

In the following sequence (Figure 6), L is moving to play with the cloth on the floor, G is playing with the doll on the table while E is preparing to change his doll (Figure 6a). As L slowly moves away from the table, E simultaneously also moves away from the table holding a nappy, while oriented towards the camera (Figure 6a). L is now standing next to the table, laying the cloth on the floor to rest another doll on it (Figure 6b). E moves toward the table and as he directs his head toward G, he places the diaper he was holding on the table, in the position previously occupied by L (Figure 6c). G looks at him, and as E quickly moves back to the initial position, on the right side of the table, G takes the nappy to use it on her doll (Figure 6d).

## Discussion

By looking at spontaneous interactions between children from 19 to 28 months at kindergarten, the current study focused on how participants initiated, constructed and maintained a shared orientation towards play activities with pretend toys. Although explorative, our results seem in line with previous reports suggesting an early onset of social pretend play (Hutt et al., 2022; Luo et al., 2022), developing over a continuum from being predominately an individual activity to progressively becoming a co-constructed endeavour. In particular, play instances similar to what Howes and Matheson (1992) defined ‘parallel social’ and ‘complementary and reciprocal’ were displayed already at 19 months by children in our data corpus, and emerging ‘cooperative” social pretend play instances appeared around 24 months. However, our micro-analytical observations shed further light on the sequential construction of such play instances, placing particular emphasis on the following aspects:

1. Co-constructed sequences appeared organised by a turn-alternation structure.
2. Turn-alternation developed through different time spans. For instance, one child’s move promptly followed a move from another child (often including partial overlapping) when inter-actions included toy foods or kitchen facilities/tools. Longer pauses in between one move and the following one appeared in nursery/doll caring interactions, e.g., when two children engaged in a nappy-changing sequence.
3. Children used embodied and material resources afforded by the sequential organisation of actions to engage in and manage social play episodes.
4. Children’s play possibilities seemed to be shaped by the participatory frameworks dynamically emerging as they moved in the play settings, mostly seeking to be spatially proximal with other children.

These findings have two major implications for a developmentally- informed understanding of how young social actors organise their participation within specifically negotiated and co-constructed frameworks. First, in a developmental phase (before the second year) in which children’s affective, social and cognitive capacities and attitudes are mostly self-oriented, our results show that there is an emerging sense of ‘the other’ as an interactional presence opening up new possibilities for actions. Peer play can therefore appear as an early socialisation practice, enabling children with emerging communicative capacities to experience how to manage their own and others’ behaviours, how to negotiate meanings and actions in the locally assembled and occasioned contexts of inter-acting. As such, the motivation and capacity to engage in playful interactions may be stemming out of early intersubjective experiences and embodied experience rather than resting on purely cognitively-driven achievements (Fantasia & Nomikou, 2022). Social play offers complex participatory configurations that young children learn to manage and navigate with increasing expertise as they develop. Further investigation into this may support our understanding of how action formation and the specific organisation of sequential environments shape participation capacities in developmentally-sensitive phases.

Second, in our data even younger participants seemed able to engage in interactive exchanges constructed over turn alternations and coordination. Because actions were so tied to the afforded function(s) of objects and room facilities, the complexity of inter-actions was also shaped by the type of different objects available and to the other participants’ actions with them. For example, the toy kitchen and its accessories (small plates, saucepans or pasta box) were used for cooking or storing/serving food; biscuits or a bread loaf were often eaten at the large round table as breakfast or lunch. This seems to suggest that pretend daily-life objects familiar to the majority of Western children, e.g., plastic knives, soap bottles or washing cloths may provide a “scaffolding” function for extended object-focused interactions since very early on. And beyond their pretence quality, these interactive sequences may sustain children’s emergent ability to engage in triadic play, socialising them to the dialogic structure of human conversation.

To conclude, this study’s contribution to the field lies in reporting spontaneous examples of sequential co-construction of play actions in a developmental phase that is rarely considered by studies on pretence, bringing further evidence to (the very limited) literature on early social play interactions with pretend objects. On a more general note, while experimental and lab-based studies on play have made significant contributions to the field, studying the development of spontaneous play engagements as they occur in the ecology of children’s life remains a challenging yet crucial task. Shifting our scientific focus from lab-based, present-or-absent play skills to process models of interactions may support a dialogical view of joint actions as co-constructed: daily interactions at home or at the kindergarten offer routines organised around formal structures, which help infants learn to become competent participants in social interactions (Fantasia & Delafield-Butt, 2023).
